# CompNet: a GUI based tool for comparison of multiple biological interaction networks

**DOI:** 10.1186/s12859-016-1013-x

**Published:** 2016-04-26

**Authors:** Bhusan K. Kuntal, Anirban Dutta, Sharmila S. Mande

**Affiliations:** Bio-Sciences R&D Division, TCS Research, Tata Consultancy Services Ltd., 54-B, Hadapsar Industrial Estate, Pune, 411 013 Maharashtra India

## Abstract

**Background:**

Network visualization and analysis tools aid in better understanding of complex biological systems. Furthermore, to understand the differences in behaviour of system(s) under various environmental conditions (e.g. stress, infection), comparing multiple networks becomes necessary. Such comparisons between multiple networks may help in asserting causation and in identifying key components of the studied biological system(s). Although many available network comparison methods exist, which employ techniques like network alignment and querying to compute pair-wise similarity between selected networks, most of them have limited features with respect to interactive visual comparison of multiple networks.

**Results:**

In this paper, we present CompNet - a graphical user interface based network comparison tool, which allows visual comparison of multiple networks based on various network metrics. CompNet allows interactive visualization of the union, intersection and/or complement regions of a selected set of networks. Different visualization features (e.g. pie-nodes, edge-pie matrix, etc.) aid in easy identification of the key nodes/interactions and their significance across the compared networks. The tool also allows one to perform network comparisons on the basis of neighbourhood architecture of constituent nodes and community compositions, a feature particularly useful while analyzing biological networks. To demonstrate the utility of CompNet, we have compared a (time-series) human gene-expression dataset, post-infection by two strains of *Mycobacterium tuberculosis*, overlaid on the human protein-protein interaction network. Using various functionalities of CompNet not only allowed us to comprehend changes in interaction patterns over the course of infection, but also helped in inferring the probable fates of the host cells upon infection by the two strains.

**Conclusions:**

CompNet is expected to be a valuable visual data mining tool and is freely available for academic use from http://metagenomics.atc.tcs.com/compnet/ or http://121.241.184.233/compnet/

**Electronic supplementary material:**

The online version of this article (doi:10.1186/s12859-016-1013-x) contains supplementary material, which is available to authorized users.

## Background

Interaction networks are a convenient way of representing the complex nature of multi-component systems. Examples of such complex systems include biological pathways, social interactions, financial markets, management systems, multiple modules in a programming language, etc. Recent emergence of systems biology has brought biological networks into focus. Such biological networks can be of various types, ranging from protein-protein interactions, gene regulatory networks, metabolic networks, microbe co-occurrence and co-inhibitory networks, etc., and can be investigated using appropriate network analysis methods [1–10]. Depending on the type of the network, variations may arise due to several internal/external factors like inheritance/evolution, environmental stress, infection, etc. Identification and interpretation of these variations are therefore crucial in understanding the respective biological system.

In addition to comparison of graph properties/metrics in form of tables or charts, identifying and comprehending the patterns of variations across different networks becomes several folds easier if provisions exist for visual comparisons, such as creation of graph layouts, overlaying of multiple networks, and interactive analysis of graph components. Several currently available methods/tools allow comparison of multiple interaction networks, the majority of which focuses on network alignment, querying, and sub-graph matching [11–14]. With increasing interests in systems biology, tools specialized for analysis of complex metabolic networks (represented in information rich graph formats like SBML) has also been developed [15]. These tools employ different algorithms to compute pair wise similarity between selected networks/paths. Although some of these tools like MIMO [15], have options for graphical visualization of outputs, in general, the network alignment and querying methods do not provide any dedicated module/options for visual comparison of multiple networks on a single canvas. On the other hand, there are several network visualization tools available to researchers, which enable easy computation and analysis of graph properties for any given network [16, 17]. However, these network visualization tools also have limitations pertaining to comparative visualization of multiple networks. Cytoscape [18], the most popular visual platform for studying biological networks, has a limited number of plugins that focus on comparing properties of multiple input networks [19–22]. Although these tools/plugins are useful in their own context, most of them have limitations with respect to visual comparison of more than two input networks. For example, although ‘network analyzer’ [16] provides tabular summaries and charts/plots depicting the graph properties of input networks, it does not allow drawing or overlaying of multiple networks/graphs on a canvas. Similarly, ‘Venn and Euler diagrams’ [23] and ‘Venndiagramgenerator’ [21] provide a comparison of different input networks in terms of constituent nodes, and by definition have limitations pertaining to the number of sets (networks) that can be visualized using such diagrams. Another Cytoscape plugin, ‘Pina4ms’ [24], though enables comparison/overlay of multiple interaction networks, is not designed for generic use. This plugin only allows comparison of a few predefined sets (or subsets) of protein-protein interactions. Other plugins of Cytoscape pertaining to network comparison are also mostly designed for network alignment and querying [25–27]. The above observations make it apparent that despite the availability of quite a few popular and comprehensive network/graph analysis tools, there remains a need for a software tool/platform that allows interactive visual comparison and analysis of multiple biological networks at the same time. In addition, the necessity for such a tool/platform can be further justified considering that biological networks exhibit certain characteristic features [28], and may occasionally require appropriate specialized comparison approaches apart from commonly used network metrics.

In this paper we present CompNet - a user-friendly GUI-based tool, which enables comparison of multiple interaction networks that are provided in the form of edge-lists, node-lists (to be overlaid on a background network), or path-lists. The tool can be used for overlaying and subsequent comparative visualization (and analysis) of multiple networks. CompNet helps to elucidate similarities/differences between the compared networks using different network metrics and visualizations, appropriately designed to highlight the topology of connections between the constituent nodes, differential shortest paths, and community distributions. CompNet intends to complement existing network analysis tools/platforms and incorporates the methods/metrics/options which would be used most frequently during multiple-network comparisons. Any further analysis with other user-preferred network analysis tools also becomes easy, given the provisions of exporting the results and networks diagrams created using CompNet into user-friendly output formats.

## Implementation

**Fig. 1:**
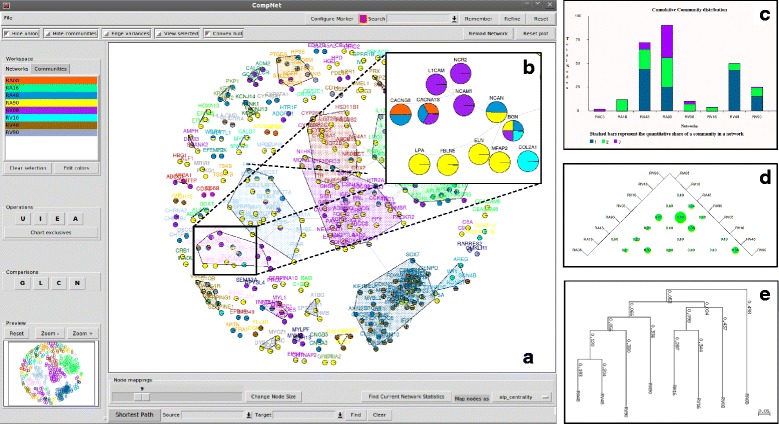
(**a**) CompNet canvas displaying the union of eight protein-protein interaction networks. The names of nodes belonging to different communities are marked with different colors. (**b**) The ‘pie-nodes’ representation enables to identify presence/absence of individual nodes across the compared networks. (**c**) The cumulative community distribution plot (**d**) Bubble chart representing similarity between networks (**e**) Hierarchical tree built using network similarity

CompNet has been developed using PerlTk and includes several graph analysis functions from the R ‘igraph’ package (http://igraph.sourceforge.net). The tool allows easy visualization of the union, intersection and/or complement regions of any selected set of networks. Different visualization features (e.g. ‘pie-nodes’, ‘edge-pie’ matrix, ‘chart summary’, etc.) aid in easy identification of the key nodes/interactions and their significance across the compared networks. The option for hierarchical clustering of networks (trees) based on constituent nodes/edges, using Jaccard similarity index, helps one to find the relative similarity between selected networks. CompNet neighbor similarity index (CNSI), a new metric for network similarity, can be used for capturing the neighborhood architecture of constituent nodes. Based on generic network properties, community composition, and shortest paths, a visual comparison of multiple networks using CompNet enables one to obtain deeper biological insights. Figure 1 provides a snapshot of the CompNet GUI and highlights few of the salient features of this tool.

Networks may be imported in CompNet by providing either (a) egde-lists, (b) path-lists or (c) a set of nodes to be overlaid on a ‘background’ network (Additional file 1: Figure S1). ‘Edge-list’ refers to a text file containing a list of node pairs (each line containing a pair of nodes). An edge is drawn in the displayed network between every set of nodes forming a pair. Path-lists are similar input files, where each of the lines in the input file contains multiple nodes in a specific order (a path). Edges are drawn in the network between every consecutive node in a given path. The third option of overlaying nodes on a background network essentially involves constructing a network by selecting only the interconnections between nodes of interest (‘overlaid nodes’) from a larger user provide network (‘background network’).

CompNet allows identification of the union, intersection and exclusive edges amongst a selected set of networks using simple GUI operations. The ‘union’ operation identifies (and displays) all the nodes and edges which are included in any of the loaded/selected networks. In contrast, the ‘intersection’ operation compares two or more selected graphs/networks to identify (and display) only those nodes and edges which are commonly present in each of the selected networks. The ‘exclusive’ feature identifies and displays the nodes and edges which are specific/exclusive to the selected networks. While rendering multiple networks on the canvas each node is represented as a ‘pie’ with differently coloured pie-slices corresponding to the source networks (Additional file 1: Figure S2). Hence, with a first glance at the canvas, a user can easily ascertain the affiliations of the nodes to any of the depicted networks. An array of other user friendly visualization options in CompNet enables the user to study the distribution of nodes/ edges across selected networks, communities and sub-graphs (Additional file 1: Figures S3 and S4).

CompNet makes the comparison of multiple networks convenient by providing a distribution of global graph properties like total nodes, total edges, density, clustering coefficient, average path length and diameter of the loaded networks (Additional file 1: Figure S5). These metrics allow to better understand how well connected are the components of the analysed network and enable assessment of its robustness and modularity [29]. A more detailed and flexible comparison can be made on the basis of node-specific properties like degree, centrality, betweenness, closeness, eccentricity and coreness, with options to map these specific graph properties as node sizes (proportionally to the selected metric) (Additional file 1: Figures S6, S7, S8, S9 and S10). Centrality measures are important in understanding the key components of any network. Very well connected nodes (which have a high degree values) in a biological network are often functionally more important [30–32]. High betweenness, on the other hand, characterizes nodes which lie in a significant number of paths connecting different parts of the network [30–32].

For a similar set of networks, like those representing time-series data or protein interactions from healthy versus diseased tissues/cells, the changes in shortest paths might provide valuable insights in understanding the biological mechanism [33]. CompNet allows the user to identify such shortest paths from multiple networks with ease, and visually trace/compare these paths (Additional file 1: Figure S11). An unweighted breadth-first search is used to calculated the shortest path between the source and target nodes using the ‘igraph’ library [34]. Additionally, shortest paths between multiple sets of sources and targets can also be computed with CompNet by providing it with two separate files containing lists of sources and targets. This feature can be utilized to perform shortest path based analyses, similar to the ‘express path’ analyses study by Karim and coworkers [33].

CompNet allows assessment of statistical significance of the network properties calculated for any network/sub-network displayed on the CompNet canvas. Users can evaluate whether the global network properties, namely network diameter, network density, clustering coefficient and average path length, are significantly different from background network. The background network can either be the union of the networks under comparison, or a user defined network. CompNet draws a large number of random sub-networks from the specified background network, the sizes of each of these random networks being equivalent to the size of the network being assessed (query network). The size and the number of random networks to be generated can also be specified by the user. The graph properties for all these ‘similar sized’ random networks are then calculated and properties of the query networks varying significantly from these ‘background’ distributions are assessed with a Z-test [35]. The results are displayed graphically with associated p-values depicting the significance of any observed variation(s). All the values can also be exported as text files for further analysis.

CompNet detects ‘communities’ in the union network (using standard ‘igraph’ library methods [34]) and colors them distinctly, and lists them in the ‘Community’ tab in a descending order of their size (Additional file 1: Figures S12, S13 and S14). CompNet also incorporates different methods to compute (and visualize) similarities between multiple networks. Pairwise Jaccard similarities [36] can be computed by considering the distribution of nodes (Eq. 1) and edges (Eq. 2) in the compared networks. A greater number of shared nodes or edges between two networks will result in higher Jaccard index values and imply a greater extent of similarity.

1 $$ Jaccard\  Node\  similarity = \frac{{\mathrm{Nodes}}^{\mathrm{A}}\ {\displaystyle \cap }\ {\mathrm{Nodes}}^{\mathrm{B}}}{{\mathrm{Nodes}}^{\mathrm{A}}\ {\displaystyle \cup }\ {\mathrm{Nodes}}^{\mathrm{B}}} $$

2 $$ Jaccard\  Edge\  similarity = \frac{{\mathrm{Edges}}^{\mathrm{A}}\ {\displaystyle \cap }\ {\mathrm{Edges}}^{\mathrm{B}}}{{\mathrm{Edges}}^{\mathrm{A}}\ {\displaystyle \cup }\ {\mathrm{Edges}}^{\mathrm{B}}} $$

Where ‘A’ and ‘B’ are the compared networks and the similarity values are computed based on the set of nodes/edges present in A and B.

CompNet incorporates a method for comparison of neighborhood similarities of the constituent nodes between the compared networks. CompNet neighbor similarity index (CNSI) (Eq. 3) can be used for capturing the neighborhood architecture of constituent nodes. Two nodes (from two compared networks) are deemed to be more similar if the lists of their immediate neighbors overlap. An overall similarity score, cumulated for all constituent nodes, is finally used to designate the similarity between two compared networks.

3 $$ CNSI = {\displaystyle {\sum}_{i=1}^N\frac{f_{n_i}^A\ {\displaystyle \cap }\ {f}_{n_i}^B}{f_{n_i}^A\ {\displaystyle \cup }\ {f}_{n_i}^B}} $$

Where n_i_ is the ‘i’th node in the union of compared networks A and B (consisting of a total of N nodes), and f_ni_^A^ and f_ni_^B^ are the first neighbors of n_i_ in the networks A and B respectively.

Based on the similarities computed between compared networks, CompNet enables generating hierarchical clustering diagrams (dendograms) [37] and bubble charts (Additional file 1: Figure S15).

## Results and discussions

### Insights into *Mycobacterium tuberculosis* infection through comparison of multiple biological interaction networks using CompNet

Tuberculosis is currently a global health problem and nearly a third of the world’s population is feared infected with the causative *Mycobacterium tuberculosis* (Mtb). However, active disease is not expressed in all infected individuals. The choice between the alternate outcomes (latent-infection/active-disease) is dictated by a complex network of interactions in the host and the pathogen. Moreover, different strains of Mtb have been observed to elicit different types of responses in the human host. Considering the multi-component nature of the human immune response, adoption of a network comparison based approach is expected to provide better insights while analyzing different infection types/conditions. In a previous study [33], a network based approach (using shortest-path comparisons) was used for identifying key regulatory nodes controlling host response during tuberculosis infection. We have used CompNet to re-analyze the time-series micro-array datasets used in this study. These datasets pertain to gene-expression of human macrophages infected with two strains (H37Ra and H37Rv) of *M. tuberculosis*. While the strain H37Rv is known to avoid the host defensive mechanisms, thereby causing persistent infection, the other strain H37Ra is an attenuated avirulent strain. Various network comparison approaches, implemented in CompNet, have been used to identify key genes and biological processes that are likely to play crucial roles during host response to Mtb infection.

In the present analysis, the gene expression data of human macrophages infected with H37Ra and H37Rv at 5 infection time-points (0, 8, 16, 48 and 90 h) were downloaded from the supplementary material provided by Karim and co-workers [33]. For every time-point, only the significantly perturbed nodes (having |expression values| > =3, i.e. showing both highly positive as well as negative perturbations) were filtered and obtained as ‘node lists’. The human STRING (version 9.0) interaction network [38], filtered with a stringent cut-off score of >900 (i.e. retaining only high confidence interactions), was loaded as a background PPI network in CompNet. Upon overlaying the node lists on this background, eight networks were obtained (the 0 h time-point was excluded since it had no significantly perturbed genes). These networks depicted the progression of host cell responses against infections by H37Ra and H37Rv strains of Mtb.

### Overlaying differentially expressed genes on the host protein-protein interaction network reveals a well coordinated host-response mechanism

To build the host-response network(s) of infection by the two strains H37Ra and H37Rv (abbreviated as ‘RA’ and ‘RV’ respectively), relevant human gene expression data [33] corresponding to four post-infection (8, 16, 48 and 90 h) time-points were considered. The sets of differentially expressed genes were identified for each time-point. The host (human) protein-protein interaction (PPI) network was suitably modified to represent a background network for overlaying the differentially expressed genes. Individual interaction networks (RA8, RA16, RA48, RA90, RV8, RV16, RV48 and RV90), representing only the most significantly perturbed interactions (involving both up-regulated and down-regulated genes), were thereby generated for each infection time-point (Additional file 1: Figure S1). This was done to ensure that the focus of the analysis was restricted to highly ‘perturbed’ but ‘connected’ components in the network, rather than the whole set of differentially regulated genes. It is imperative that the connected nodes/proteins in the PPI networks represent some biological function brought about by the coordinated effort of multiple genes/proteins. It also needs to be considered here that host response is not instantaneous, but a prolonged and well orchestrated event. Genes/proteins perturbed at one time-point can affect its neigbouring genes/proteins (in the interaction network) at subsequent time-point(s). Therefore a union of all the individual time-point and infection-type specific networks consisting of perturbed nodes (and their known inter-connections) was constructed to obtain an overall view of the host cell machinery responding to the infection. It may be noted here, that although creation of such a ‘union’ network, and subsequently drawing any inferences from its analysis, may seem inappropriate (given the different types of infections), it needs to be considered that each of the connections in this ‘union’ network represent ‘known’ protein-protein interactions (high confidence interactions from STRING) in the host cell. Creating a ‘union’ therefore allows not only to obtain an overall view of the host response (independent of the infection-type), but also to identify the sets of nodes/interactions which lie in the interface of the two types of infection-specific responses. Furthermore, finding communities/modules and attributing potential functional roles to them seems more appropriate in an expectedly ‘larger’ and ‘dense’ union network than in relatively ‘smaller’ infection-type/time-point specific networks. The contribution of such communities/modules in host response against/at a specific infection-type/time-point can subsequently be investigated by checking the affiliations of the constituent nodes/edges to any of the ‘smaller networks’.

When the significantly up-regulated/down-regulated genes were considered, a total of 358 nodes (representing genes/proteins) connected by 609 edges (representing interactions between the genes/proteins) were observed in the union of all the networks (Additional file 1: Figure S2). A closer look into the network statistics using CompNet (see Methods) revealed that the union network had a significantly (*p* < 0.05) higher network density (0.010) and clustering coefficient (0.501) as compared to random networks of similar sizes (mean network density = 0.001, mean clustering coefficient = 0.263 computed for 10,000 random networks), drawn from the same background network. The average path length of the union network was further observed to have a significantly high value of 5.808 (*p* <0.05), in contrast to what could be expected for a network having similar size (mean of average path lengths of 10,000 random networks = 2.867). These results suggest that the genes exhibiting perturbed expression during infection are more densely connected to each other, than any randomly chosen set of genes/proteins in the background (human) PPI network, thereby suggesting a well coordinated host response mechanism during infection. A detailed analysis of network properties, while cumulating the infection type specific networks (across all time points) into separate ‘union’ networks, was also performed. These results also echoed the earlier observations pertaining to significantly higher network density and clustering co-efficient as compared to random networks of similar sizes (Additional file 1: Table S1).

### Indication of central nodes (genes) to be involved in immune-regulation, cell proliferation and cell death

Additional file 1: Figures S6 and S7 shows the top 10 nodes, in terms of betweenness and degree, in the overall union network (containing both up-regulated and down-regulated genes across four infection time-points by the two Mtb strains). The colored stacks in the bar-plots represent the qualitative presence of a gene/node in the individual networks. The height of individual stacks in the plot indicates the value of the selected graph property (e.g., betweenness, degree, etc.). As evident from the figure, the B1RC5 gene was seen to have the highest betweenness as well as the highest degree and was observed to be present in the networks corresponding to 48 and 90 h post infection by both H37Ra and H37Rv (i.e., RA48, RA90, RV48 and RV90). KCNJ11 and BUB1B were identified as the nodes having the second highest values of betweenness and degree respectively, and were found to be present in the networks corresponding to the late infection time-point (90 h) for both H37Ra and H37Rv infections. While the gene KCNJ11 codes for a membrane protein, BIRC5 and BUB1B are known to play active roles in promoting cell proliferation, progression of mitosis and prevention of apoptosis [39–43]. Interestingly, both BIRC5 and BUB1B were observed to be significantly down-regulated during the late infection stages (48th and 90th hour time-points) in both H37Ra and H37Rv infected macrophages. This observation leads to the question as to whether apoptosis could be the probable fate of both types of infected cells. Results obtained during a subsequent community analysis (see next section) however indicate that a higher rate of apoptosis is induced in case of infection with H37Ra cells. When the H37Ra and H37Rv infection specific host response networks were separately analysed (i.e. one union network consisting RA8, RA16, RA48, RA90, and another union network consisting of RV8, RV16, RV48, RV90, respectively), similar sets of central nodes (Additional file 1: Figure S8), as compared to those found in the overall union network, were identified. Genes like BIRC5, KCNJ11, INS-IGF, SOCS3 and FOXA2 were observed to have high betweenness in the union of host response networks against H37Ra infection, and were present exclusively in the networks corresponding to later time points of infection. These genes have been reported earlier to be associated in inducing apoptosis [39–41, 44–46]. Furthermore, except BIRC5, all of these genes were found to be upregulated during H37Ra infection. BIRC5, as mentioned earlier, is a negative regulator of apoptosis, and based on these observations it may be expected that a higher rate of apoptosis is induced in case of infection with H37Ra cells. In contrast, a majority of central nodes identified in the union of host response networks against H37Rv infection, which includes CCNA2, BIRC5, CHEK1, CDC6 and E2F1, were found to be downregulated during late infection time-points. Given that these genes also have reported roles in regulating apoptosis [39, 42, 47–50], the observations are indicative of an alternate outcome of infection with H37Rv as compared to H37Ra infection.

While analyzing the distribution of up-regulated and down-regulated genes (nodes) in different host response networks, up-regulation of 231 genes were found to be exclusive to either H37Ra or H37Rv infected cells. In contrast, only 25 of the down-regulated genes were found to be exclusively present in either H37Ra or H37Rv infected host response networks. Subsequently host response networks consisting of significantly up-regulated and down-regulated genes were separately constructed and analysed. As expected (from the distribution patterns of up-/down-regulated genes), the networks consisting of up-regulated host genes could discriminate better between response to H37Rv and H37Ra infections. Additional file 1: Figure S9 depict the union of the ‘up-regulated’ host response networks (for different time-points), highlighting the degree and betweenness of individual nodes. The ‘pie-nodes’ representations also depict the association of each of the genes to different infection time-points, thereby allowing easy identification of time-point specific mediators (genes) of host response. In contrast to the observation mentioned in the previous paragraph, a new set of nodes having high values of degree and betweenness were identified (Additional file 1: Figure S10 represents the top 10 perturbed nodes). These genes included INS-IGF2 (an auto antigen that causes auto immunity and cell death [44]), SOCS3 (a suppressor of cytokine signaling [45]), CCR5 (known to be an important co-receptor for macrophage-tropic virus, including HIV, facilitating entry into host cells [51]), IFNG (having antiviral, immunoregulatory and anti-tumor properties and a potent activator of macrophages [52]) and IL17A (a pro-inflammatory cytokine produced by activated T cells [53]). The stacks in the bar plots (Additional file 1: Figure S10) and the coloured slices of the pie-nodes (Additional file 1: Figure S9) further indicate that most of the nodes from this new set are exclusive to networks corresponding to infection by the H37Ra strain. The genes INS-IGF2, SOCS3, CCR5, IFNG and IL17A, known to be involved in immune response, are found to be specific to the networks corresponding to H37Ra infected cells. This observation indicates stronger host response to infection by H37Ra as compared to that by the H37Rv strain.

### Community analysis reflects differences in host response during progression of infection by virulent and avirulent strains

**Fig. 2:**
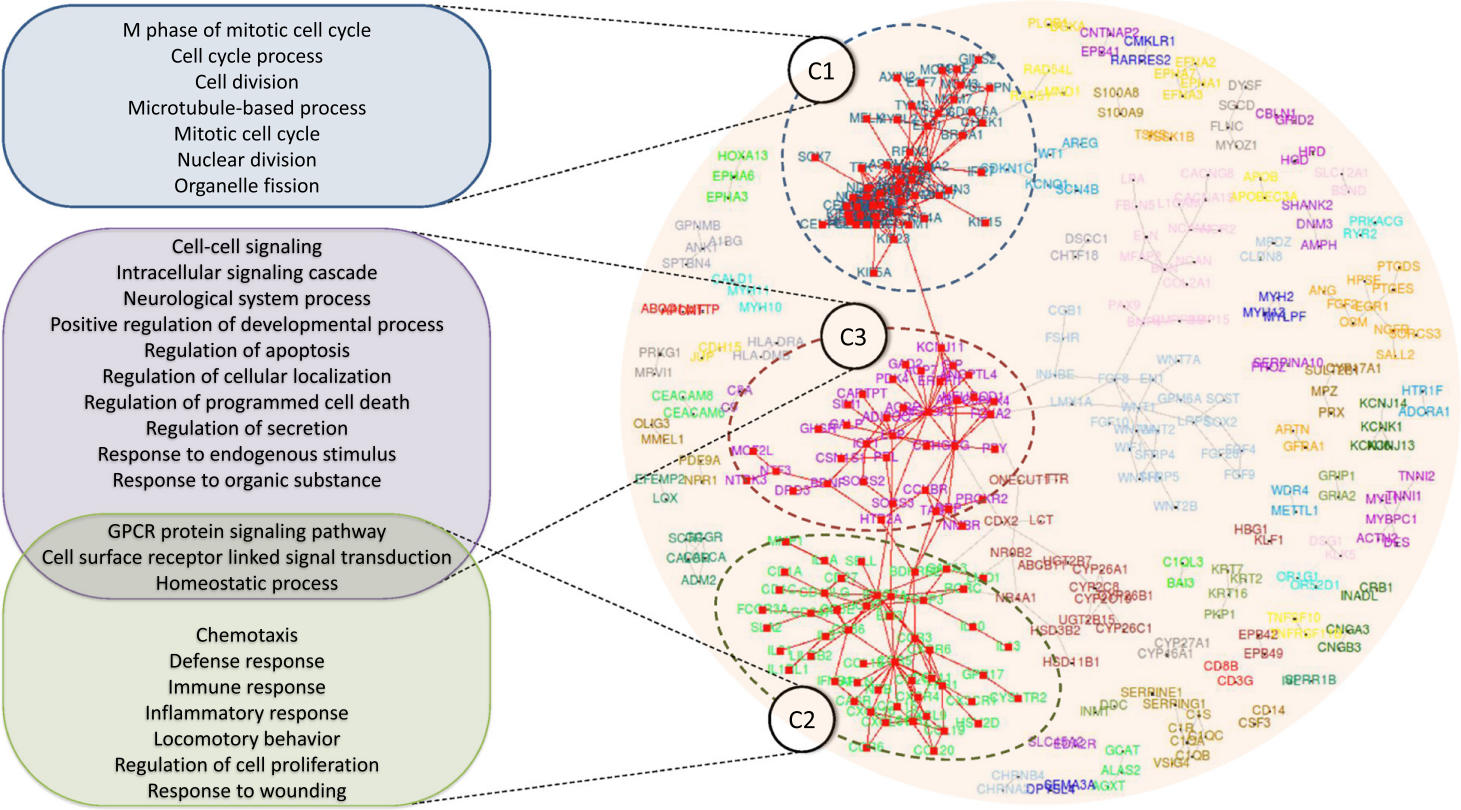
The 3 largest communities in the union network, identified by CompNet. GO Biological process terms which were found to be enriched in these communities are highlighted. The nodes/interactions in these communities correspond to some distinct, but related biological processes. While the nodes constituting community C1 are mostly involved in regulation of cell cycle and cell division, the other two communities (C2 and C3) participate in various cellular signaling processes, inflammation and chemotaxis. The members of the community C3, in addition to cell signaling, are also involved in processes like secretion, cell death and apoptosis. (To avoid redundancy, some similar GO Biological process terms are not shown in the figure)

Inferring the fate of infection from the expression levels and connections between individual nodes in the network may not be sufficient for a complete understanding of the complex biological system. To get a deeper insight, the analysis was further extended to detect closely connected communities/modules in the union network and subsequently analyze their functional participation. A total of 65 such modules were identified from the union network (consisting of both up-regulated and down-regulated genes) using the ‘fast-greedy’ community detection algorithm (default option in CompNet) [54]. The 3 largest communities, referred to as ‘C1’, ‘C2’ and ‘C3’ (Fig. 2), constituted of 142 nodes densely connected with 412 edges. Closely knit communities of genes are expected to contribute to related biological processes/pathways [55]. To investigate such functional aspects of the identified communities, the constituent nodes of C1, C2 and C3 were selected from the CompNet canvas for performing an ontology enrichment study (GO enrichment) using the DAVID tool [56, 57]. A formatted output showing the biological processes associated to the three communities is shown in (Additional file 1: Table S2). While the nodes constituting community C1 are mostly involved in regulation of cell cycle and cell division, the other two communities (C2 and C3) participate in various cellular signaling processes, inflammation and chemotaxis (Fig. 2). Interestingly, members of the community C3, in addition to cell signaling, are also involved in processes like secretion, cell death and apoptosis.

The cumulative community distribution profile, plotted using CompNet, indicated some interesting results (Additional file 1: Figure S12). For example, the number of nodes/edges present in these communities varied significantly across the individual networks (representing the different time-points post-infection). The number of ‘differentially regulated’ nodes, constituting these communities, steadily increased till the 48th hour time-point for both H37Ra and H37Rv infected cells. For both types of infection, the maximum perturbation at the 48th hour time-point was observed in community C1, which was the largest community in the union network. Additional file 1: Figure S13 depicts the intersecting edges between the networks RA48 and RV48. As one would expect, it was observed that a majority of the intersecting edges belonged to the community C1. The members of the community C1, when visualized as an ‘edge-pie’ matrix plot (depicting the edge distribution across the networks), further revealed that almost all of the interactions from community C1 for the 48th hour time-point were common for both H37Ra and H37Rv infected cells (Additional file 1: Figure S14). This observation can probably be attributed to some defense mechanism commonly employed by the human cell against both H37Ra and H37Rv infection.

It was also interesting to note that the total number of interactions involving differentially regulated genes significantly reduced in H37Rv infected cells at the 90th hour time-point. Only a slight increase in the number of perturbed nodes in community C2 could be spotted for H37Rv infected cells. In contrast, for the H37Ra infected cells, the total number of interactions involving perturbed genes further increased at this late time-point post-infection. Additional perturbations in the latter case could also be identified in communities C2 and C3.

Increase in perturbations in the community C2, expected to be associated with enhanced activity of the cell signaling and inflammation pathways, could be noticed for infections by both H37Ra and H37Rv. However, increased number of differentially regulated genes in community C3 was found to be exclusive to H37Ra infection. As mentioned earlier, GO enrichment analysis indicated that regulatory paths for several biological processes like programmed cell death, apoptosis, and secretion were associated to this community. This observation seems to be consistent with earlier studies which indicated that H37Ra infection induces apoptosis to a much higher degree than infection by the H37Rv strain [58–60].

The gradually increasing perturbations in case of H37Ra infection probably pertains to the continued efforts of the host cell towards neutralizing the avirulent strain. On the other hand, the initial increase and subsequent reduction in the number of differentially regulated genes, observed in case of H37Rv infection, probably points at the pathogen evading the host defense systems, thereby proceeding towards a persistent infection.

### Inferring similarity between host response networks by comparing node-neighbourhoods

CompNet incorporates a method for comparison of multiple interaction networks on the basis of neighbourhood similarities of the constituent nodes. Two nodes (from two compared networks) are deemed to be more similar if the lists of their immediate neighbours overlap. An overall similarity score (called CNSI or CompNet neighbour similarity index), cumulated for all constituent nodes, is finally used to designate the overall similarity between any two compared networks. The eight networks corresponding to the different infection time-points by H37Ra and H37Rv were compared using this method. The results of the comparison, in the form of a bubble chart and a dendogram (Additional file 1: Figure S15), depicted grouping of the different networks according to their similarities. At a first glance, the bubble plot of ‘similarity profile’ between the networks showed that the networks corresponding to the 48th hour time-point post infection by both H37Ra and H37Rv had the highest CNSI (represented by the largest bubble on the chart). The dendogram placed the networks corresponding to the late infection time-points (48 and 90 h post infection) in a single separate cluster, indicating their similarity as compared to the early infection time-points (8 and 48 h). A closer look into this clustering also revealed that while the host response at the 48th hour time-point was similar for both types of infection, the response for H37Ra infection at the 90th hour time-point was well separated from other late infection time-points. This observation could probably be attributed to the aggravated host response to H37Ra infection at the 90th hour time-point and is in line with the expected outcome. In summary, a clear grouping of networks during early and late infections is evident from the CNSI-based grouping of networks. Also, the distinct nature of the network corresponding to 90th hour time-point post H37Ra infection probably pertains to the relatively intense host response against the avirulent H37Ra strain.

The dataset used for the current case study had been originally analysed by Karim and co-workers, using ‘express-path analysis’ [33], which essentially involved identifying ‘shortest paths’ (in a gene-/protein-interaction network) enriched with nodes (genes) showing the most-perturbed gene-expression values. These paths can be expected to control the alternate outcomes of virulent/avirulent infections through gene-regulation/protein-protein interaction events. This previous study had identified the ‘Tyrosine kinase SRC regulon’ to play an important role during Mycobacterial infections. The shortest path finding/analysis feature of CompNet can be used to easily replicate the ‘express-path analysis’ on the chosen dataset. The current case-study aimed at analysing the data from a different perspective, and to highlight how different network characteristics (e.g. centralities, community structures and neighbour similarities) when viewed in combination with the gene-/protein-functions, can help understand the infection outcomes. Since the perspectives and approaches adopted in the current case-study differ from the original ‘express-path’ analysis, the scope of comparing results is limited. However, it may be noted that the alternate outcomes of infection by H37Ra and H37Rv strains could be successfully inferred using both the earlier and the current approach. Furthermore, genes/proteins identified to be involved in host response, using the two different approaches, were observed to have similar functional profiles. For example, Karim et al. [33] identified ‘immune responses’ and ‘gene regulation’ to be the major functional classes of genes showing discrete regulation between H37Ra- and H37Rv-infected cells. In the current case-study using CompNet, communities in the union (host response) network, associated to ‘inflamation pathways’, ‘cell signalling’, ‘secretion’, and ‘programmed cell death’, were observed to be differentially contributing to the late time-point specific networks corresponding to H37Ra and H37Rv infections.

## Conclusions

The varying numbers of ‘connected perturbations’, identified by CompNet, helped in ascertaining the key components involved in host response against the avirulent H37Ra and the virulent H37Rv strains of *M. tuberculosis*. The gradually increasing perturbations in case of H37Ra infection probably pertains to the continued efforts of the host cell towards neutralizing the avirulent strain. On the other hand, the initial increase and subsequent reduction in the number of differentially regulated genes, observed in case of H37Rv infection, probably points at the pathogen evading the host defense systems, thereby proceeding towards a persistent infection. However, the observations made in this case study pertain to only a selected subset of significantly perturbed genes/interactions, and therefore require cautious interpretation. The primary objective of the present study was to demonstrate the ease with which multiple network comparison (in this case pertaining to host response at different infection time points against different strains of *M. tuberculosis*) can be performed with CompNet in order to draw biologically relevant inferences. Inclusion of experimental data at more time-points as well as with additional strains of Mtb (including MDR and XDR strains) will be useful in similar network based studies and likely to help in unraveling of newer perspectives on Mycobacterial infection.

CompNet is a user-friendly tool which allows simultaneous visualization and comparison of multiple networks. In addition to computing generic graph properties for individual networks, the tool allows multi-graph comparisons and similarity based grouping of networks. CompNet also allows visual identification and selection of sub-graphs/communities of interest, enabling a general user to work with and compare between sufficiently complex and large networks. In this work we have demonstrated how CompNet can be used to perform different analyses with multiple biological networks in order to obtain meaningful insights. Inspite of having several generic, as well as, specialized plug-ins for network analysis, the popular network analysis platforms like Cytoscape have limited user friendly options pertaining to comparison/visualization of multiple networks on the same canvas. CompNet intends to fill in this particular gap and make ‘multiple network comparisons’ easy. It may however be noted that the network analysis/comparison operations, and most of the metrics computed by CompNet, comprise only a subset of all possible network analysis methods. Encompassing all of these methods/techniques into a single platform, being a Herculean task, can be best addressed by community supported development e.g. Cytoscape plugins. Given this, CompNet only includes the methods/metrics/options which would be used most frequently during multiple-network comparisons, while keeping options open for the user to export the networks/data from CompNet into other user-preferred tools (like Cytoscape) for further analysis. It may be noted here that designing CompNet as a Cytoscape-plugin has not been considered in order to avoid dependency and portability issues associated with Cytoscape (and Java) versions [61]. However we acknowledge the ample number of visualization options in Cytoscape along with its different useful plugins. In view of this, CompNet provides options for easy export of networks to Cytoscape compatible formats (GML and edge-lists). CompNet is expected to be a valuable tool for biologists and other researchers working in the field of visual data mining.

### Ethics approval and consent to participate

Not applicable.

### Consent for publication

Not applicable.

### Availability of data and materials

**Project name**: CompNet

**Project home page**: http://metagenomics.atc.tcs.com/compnet/ or http://121.241.184.233/compnet/

**Operating system(s)**: Linux and Windows (32 and 64 bit)

**Programming language**: PerlTk

**Other requirements**: R with igraph package

**License**: Not applicable (freely available for academic and non-commercial use)

**Any restrictions to use by non-academics**: Restricted from commercial use without prior consent.

### Endnotes

Not applicable.

## Additional file

Additional file 1: This file contains supporting figures and tables relevant to the 'Implementation' and 'Results and Discussion' sections of the manuscript. (PDF 1.24 mb)

## Abbreviations

CNSI: CompNet neighbor similarity index
GML: graph modeling language
GO: gene ontology
GUI: graphical user interface
H37Ra: H37Ra strain of mycobacterium tuberculosis
H37Rv: H37Rv strain of mycobacterium tuberculosis
MDR: multi drug resistant
Mtb: mycobacterium tuberculosis
SBML: systems biology markup language
XDR: extreme drug resistant
