# Correction to: CompNet: a GUI based tool for comparison of multiple biological interaction networks

**DOI:** 10.1186/s12859-019-3168-8

**Published:** 2019-11-20

**Authors:** Bhusan K. Kuntal, Anirban Dutta, Sharmila S. Mande

**Affiliations:** Bio-Sciences R&D Division, TCS Research, Tata Consultancy Services Ltd, 54-B, Hadapsar Industrial Estate, Pune, Maharashtra 411 013 India

**Correction to: BMC Bioinformatics (2016) 17:185**

**https://doi.org/10.1186/s12859-016-1013-x**

Following publication of the original article [1], the authors requested to update a link in the article. There was a server update and the hosted applications needed to move to a new web location. The link of CompNet above published paper has changed and therefore needs to be updated in the BMC site.

The links that needs to be updated can be find at the Conclusions section of Abstract and other under Project home page at the bottom.

Old link (s):

http://metagenomics.atc.tcs.com/compnet/ or

http://121.241.184.233/compnet/

Updated new link:

https://web.rniapps.net/compnet/
