# Supplementary material for: CompNet: a GUI based tool for comparison of multiple biological interaction networks
Source: BMC Bioinformatics. 2016 Apr 26;17:185. doi: 10.1186/s12859-016-1013-x (PMC4845442; doi:10.1186/s12859-016-1013-x)
Supplement: Additional file 1: — This file contains supporting figures and tables relevant to the 'Implementation' and 'Results and Discussion' sections of the manuscript. (PDF 1.24 mb) [file 12859_2016_1013_MOESM1_ESM.pdf]

Figure S1. **(A)** Networks can be loaded in CompNet in 3 ways : (1) directly as 'edge-lists', (2) by building sub networks from a base/background network, or (3) by loading a list of paths (series of edges). **(B)** For the current case study, eight node-lists corresponding to perturbed human genes (from macrophages infected by H37Ra and H37Rv strains of *M. tuberculosis,* during four post infection time-points) were overlaid on a background human protein-protein interaction network. Eight networks were thus obtained, which were further analysed. **(C)** While building networks directly from edge-lists, CompNet allows the users to specify field delimiters and designate which of the columns in the input file corresponds to the nodes, edge-weights, or any other properties.

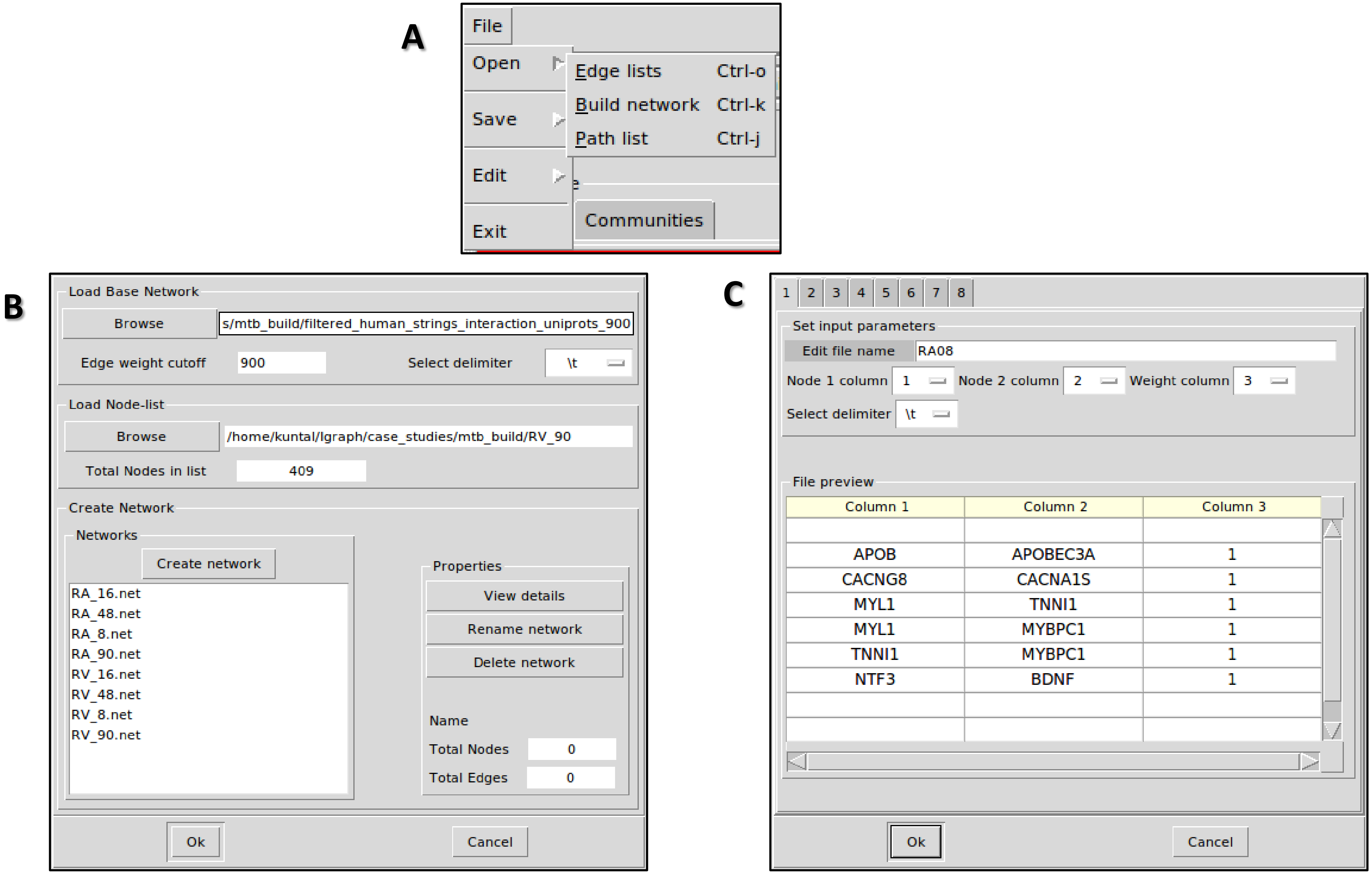

Figure S2. CompNet canvas displaying the union of eight networks (RA8, RA16, RA48, RA90, RV8, RV16, RV48 and RV90), corresponding to human macrophage cells infected by two strains of *M. tuberculosis* (H37Ra and H37Rv) at four different time-points (8, 16, 48 and 90 hours) post infection (see Results section of the manuscript for details). The node-labels belonging to different communities/modules in this union network are marked with different colors, while the nodes themselves are represented in form of pie-charts. The color of the slices of the 'node-pies' indicate the presence/absence of individual nodes across the compared networks. It is worthy to note that, several of the perturbed genes (nodes) in this network are densely connected to each other in the background network, thus forming several closely knit communities.

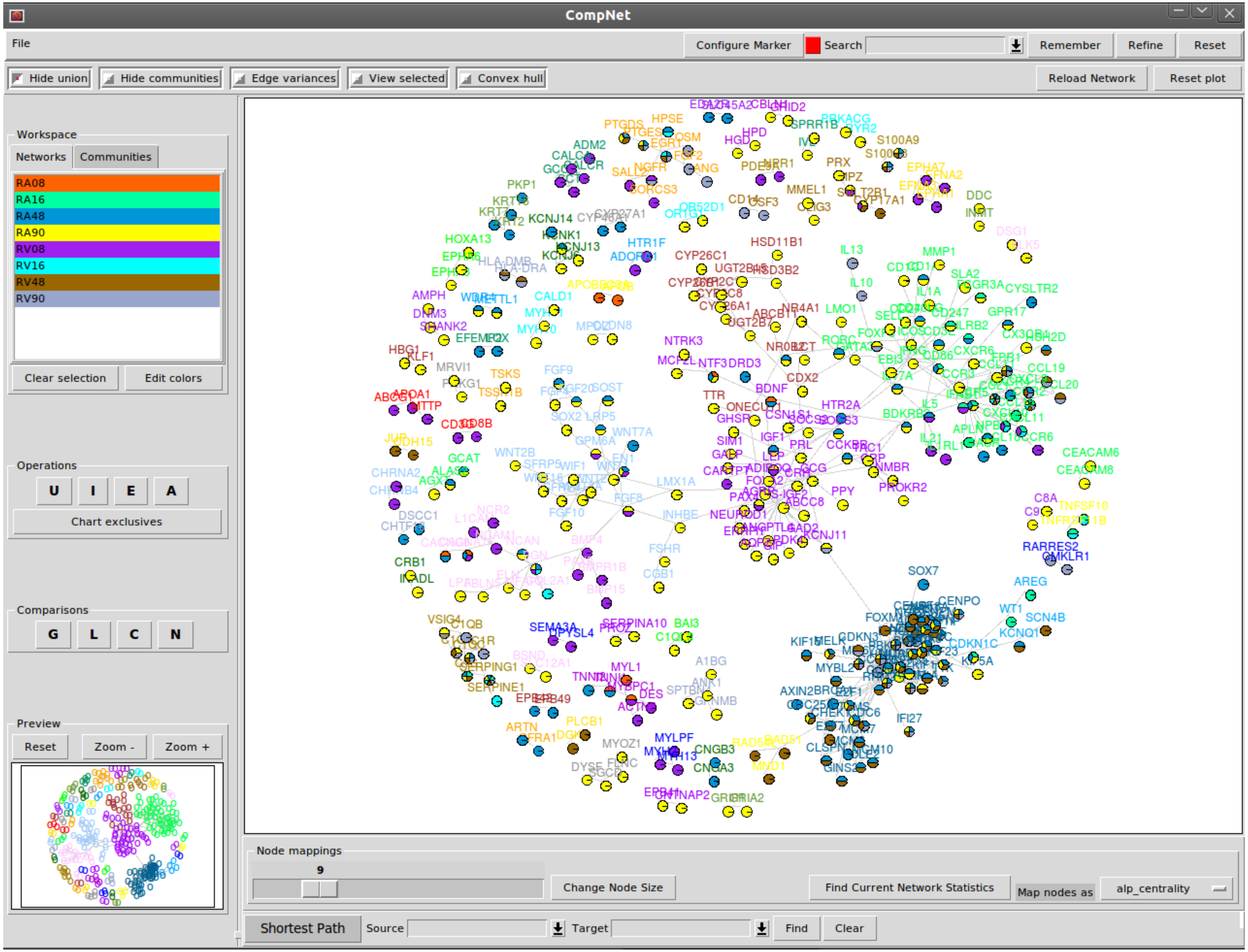

Figure S3. Visualization summary of exclusive edges across the loaded networks using the 'Chart exclusives' feature. Edges present across various combinations are highlighted using the assigned colors for each network. The upper plot margin displays the member network count for each combination in a sorted order. The lower margin of the plot displays the count of the total exclusive edges for each combination along with an interactive bar chart. Left clicking on each bar displays the corresponding edges for the selected combination as a sub-network in the main CompNet canvas with the detected communities in the background.

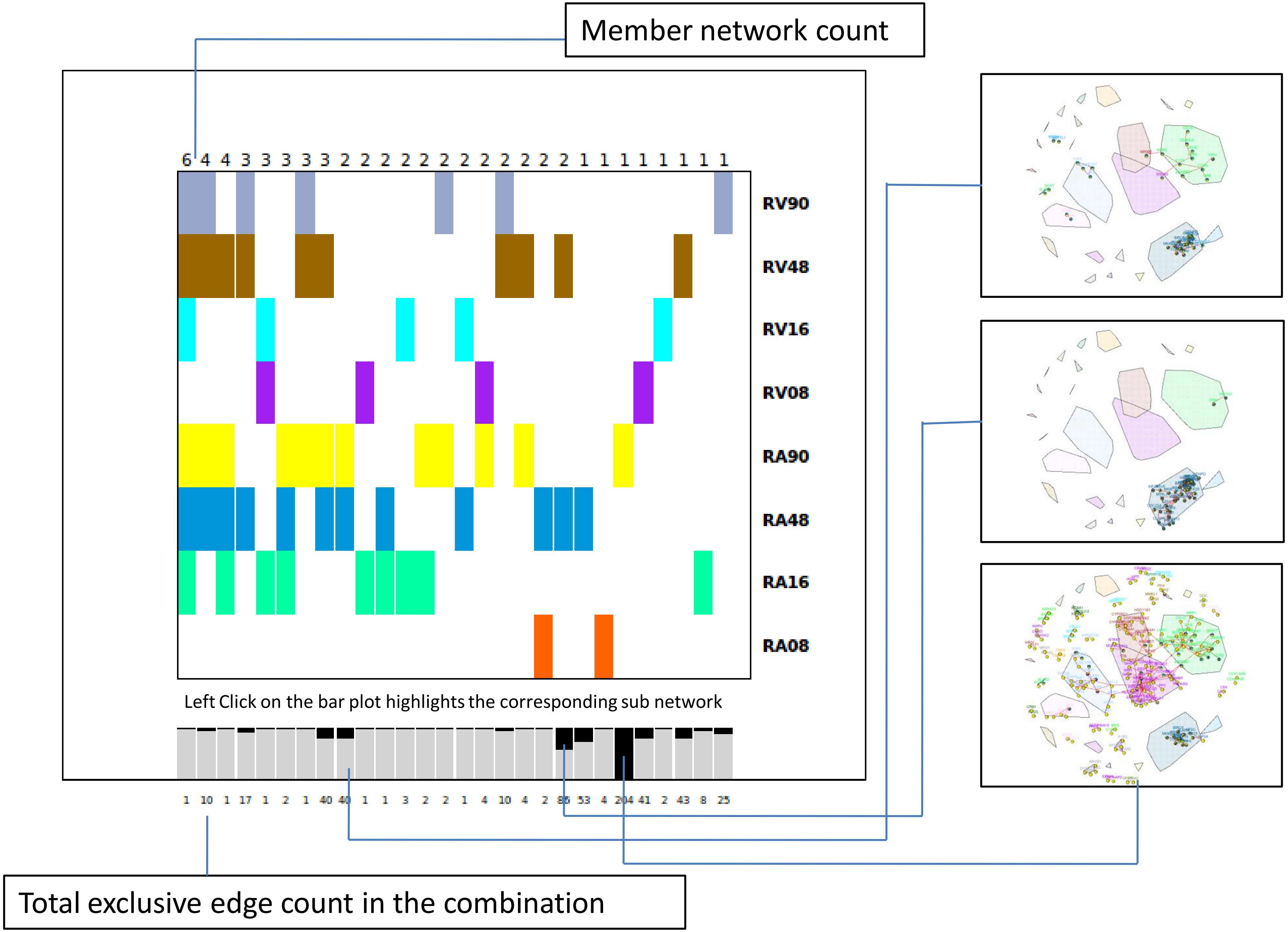

Figure S4. Visualizing pie-nodes, edge variance and edge presence (edge-pie chart). In order to visualize the qualitative presence of edges across networks for a selected set of nodes, an 'edge-pie' matrix plot can be generated for a selected set of nodes. The edges are colored according to the variance of the node presence across the loaded networks.

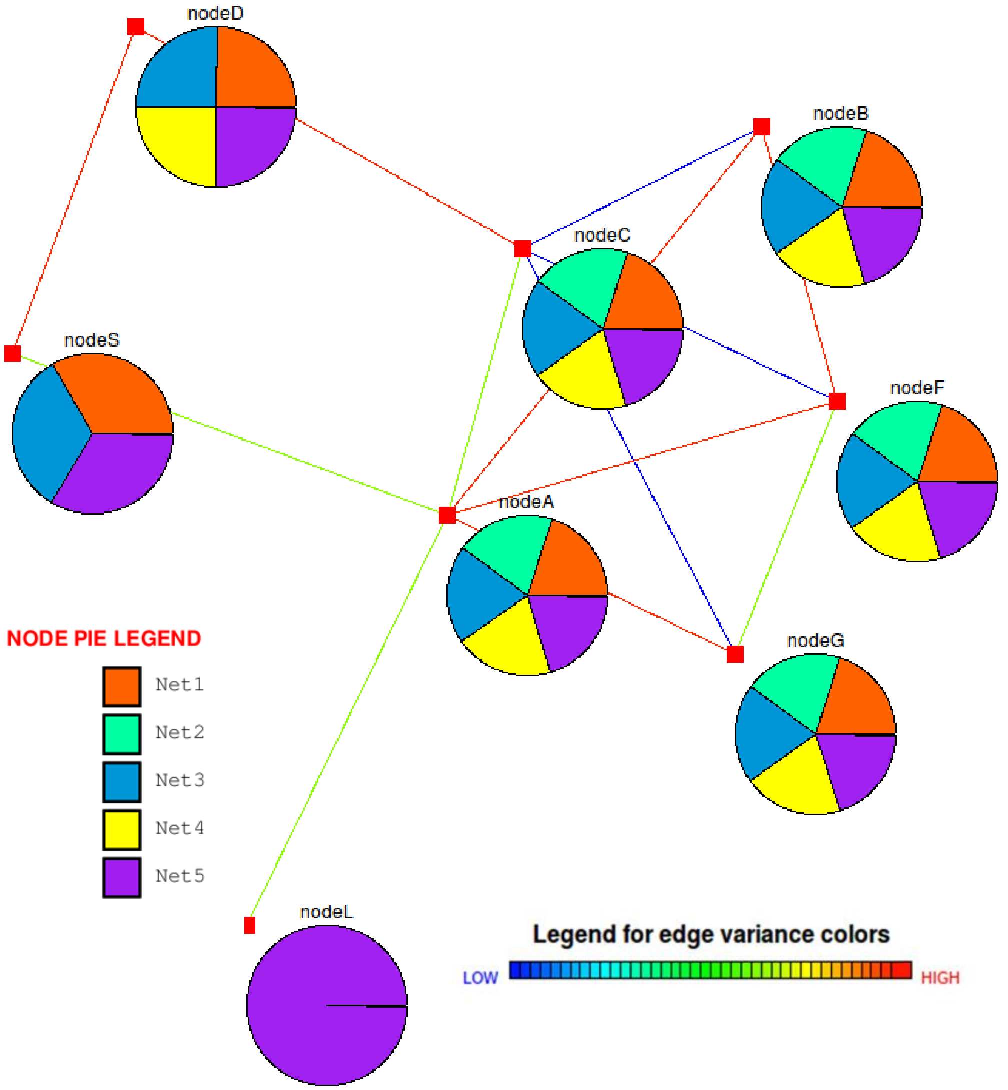

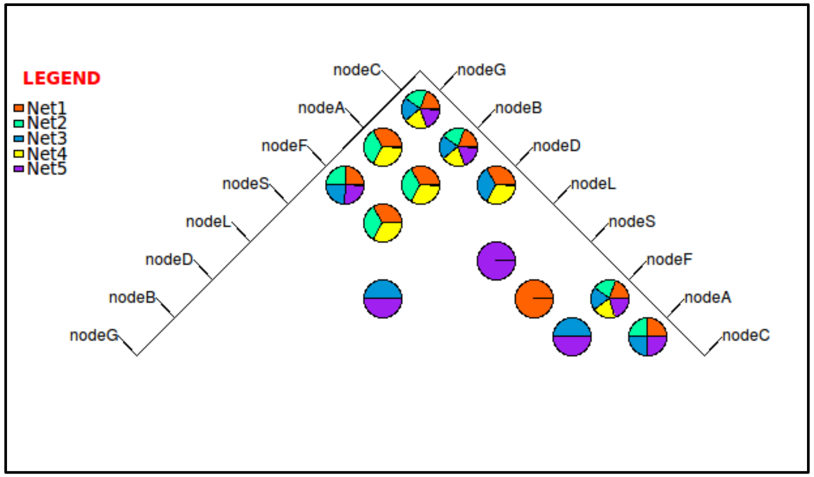

Edge-pie matrix plot

NODE	Net1	Net2	Net3	Net4	Net5
nodeF	1	1	1	1	1
nodeB	1	1	1	1	1
nodeD	1	0	1	1	1
nodeL	0	0	0	0	1
nodeG	1	1	1	1	1
nodeA	1	1	1	1	1
nodeC	1	1	1	1	1
nodeS	1	0	1	0	1

Node presence summary across the networks

Figure S5. Visualizing global graph properties using CompNet. Each tab in the plot corresponds to a global graph property. The calculated property values can be exported as a tab delimited table for future reference.

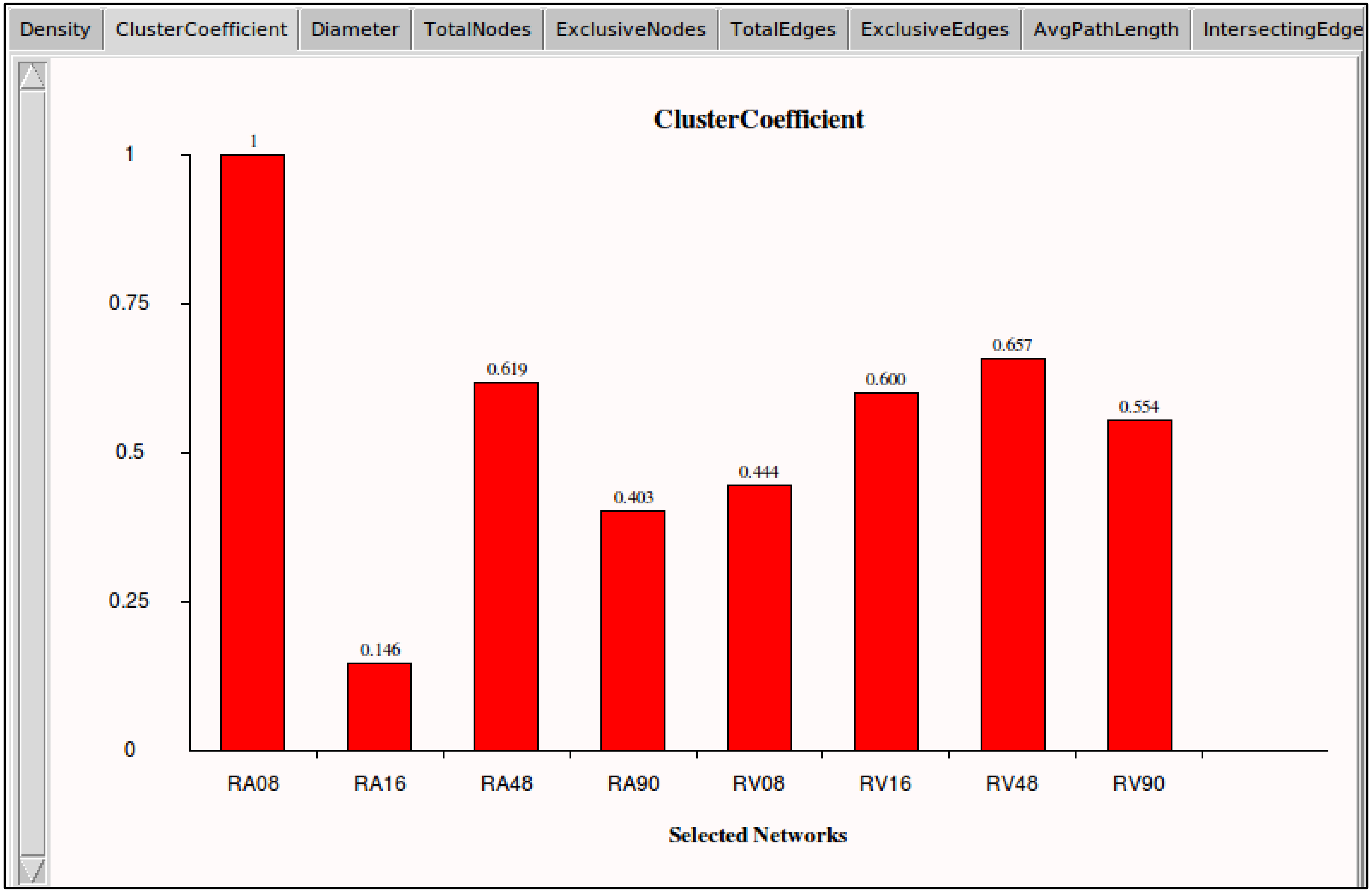

Figure S6. Graph properties of the union network, depicting both upregulated and downregulated host genes/proteins during infection by H37Ra and H37Rv strains of *M. tuberculosis*, are displayed as a cumulative bar plot using CompNet. The top 10 nodes (in terms of the evaluated graph metrics) are shown in the respective bar plots. The coloured stacks indicate the presence of the nodes in different networks. The B1RC5 node has the highest betweenness as well as degree and is present in the networks corresponding to the 48th and 90th hour post-infection time points for infection by both H37Ra and H37Rv strains (RA48, RA90, RV48 and RV90). KCNJ11 and BUB1B are the second highest nodes in terms of betweenness and degree respectively and are present in the networks corresponding to the late infection time-points of (for both types of infection).

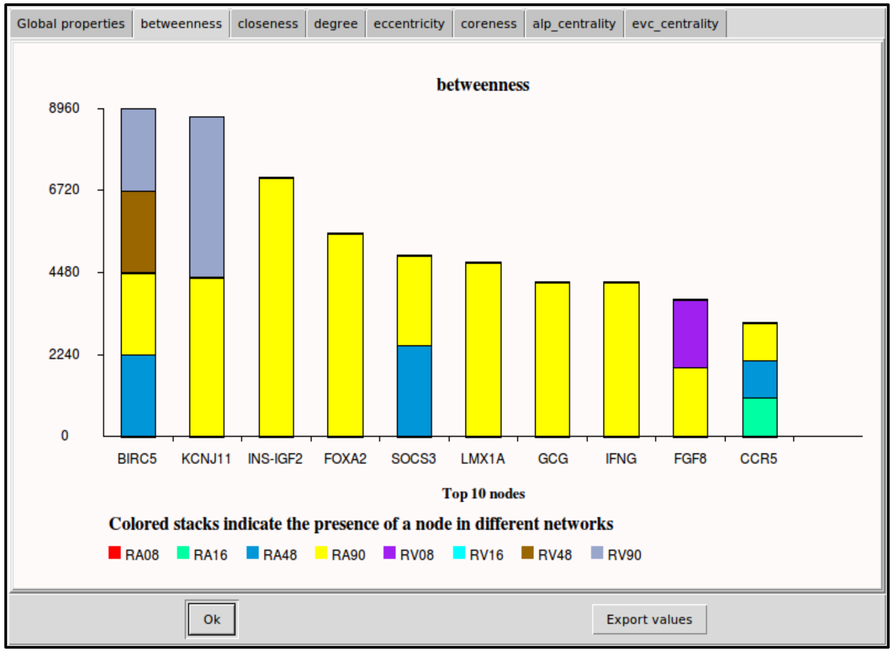

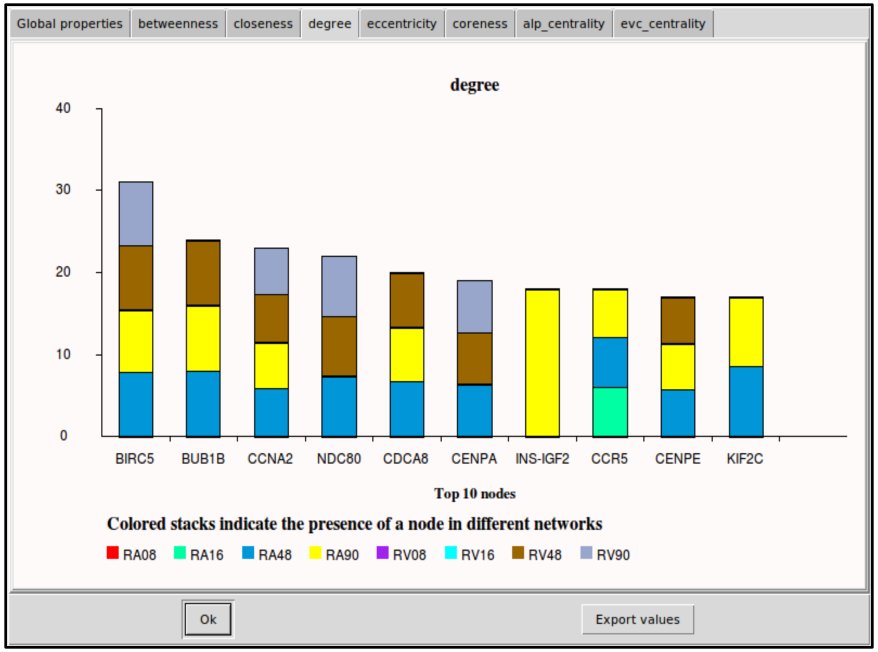

Figure S7. A view of the union of eight networks (RA8, RA16, RA48, RA90, RV8, RV16, RV48 and RV90), corresponding to host response to infections by two stains of *M. tuberculosis* (H37Ra and H37Rv), depicting the highly up-regulated genes at four different time-points (8, 16, 48 and 90 hours) post infection. CompNet has been used to highlight the degree of the nodes by mapping the node-radii to degree values. The presence of a node in a particular network (corresponding to the infection type/time-point) is also discernable from the colours of the 'pie-slices'. Nodes INS-IGF2 and CCR5 have high degree and are seen to be exclusively present in the networks corresponding to infection by the H37Ra strain (at 16th, 48th and 90th hour time-points).

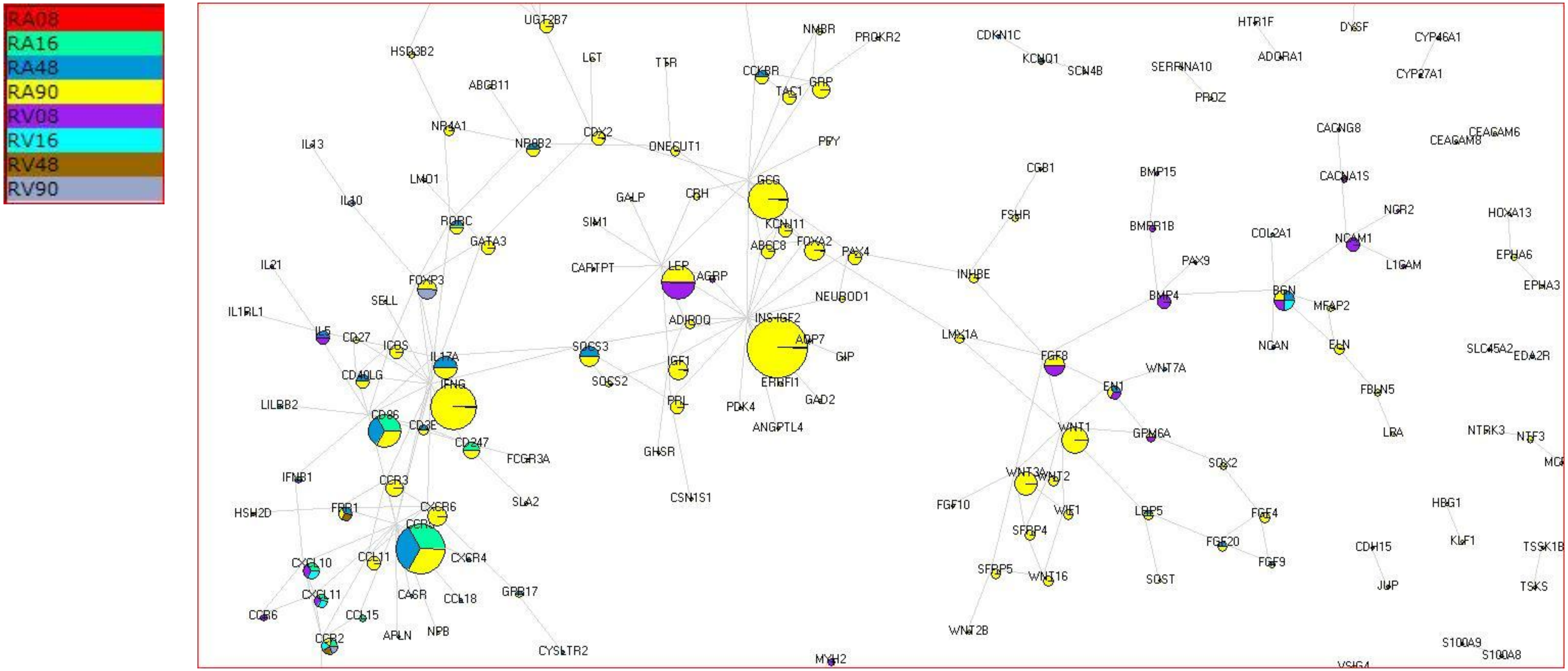

Figure S8. Graph properties (degree and betweenness) of host response networks against *M. tuberculosis* infection. (A) Graph properties of the union of host response networks against H37Ra. (B) Graph properties of the union of host response networks against H37Rv. The Graph properties are displayed as a cumulative bar plot using CompNet. The top 10 nodes (in terms of the evaluated graph metrics) are shown in the respective bar plots. The coloured stacks indicate the presence of the nodes in different networks.

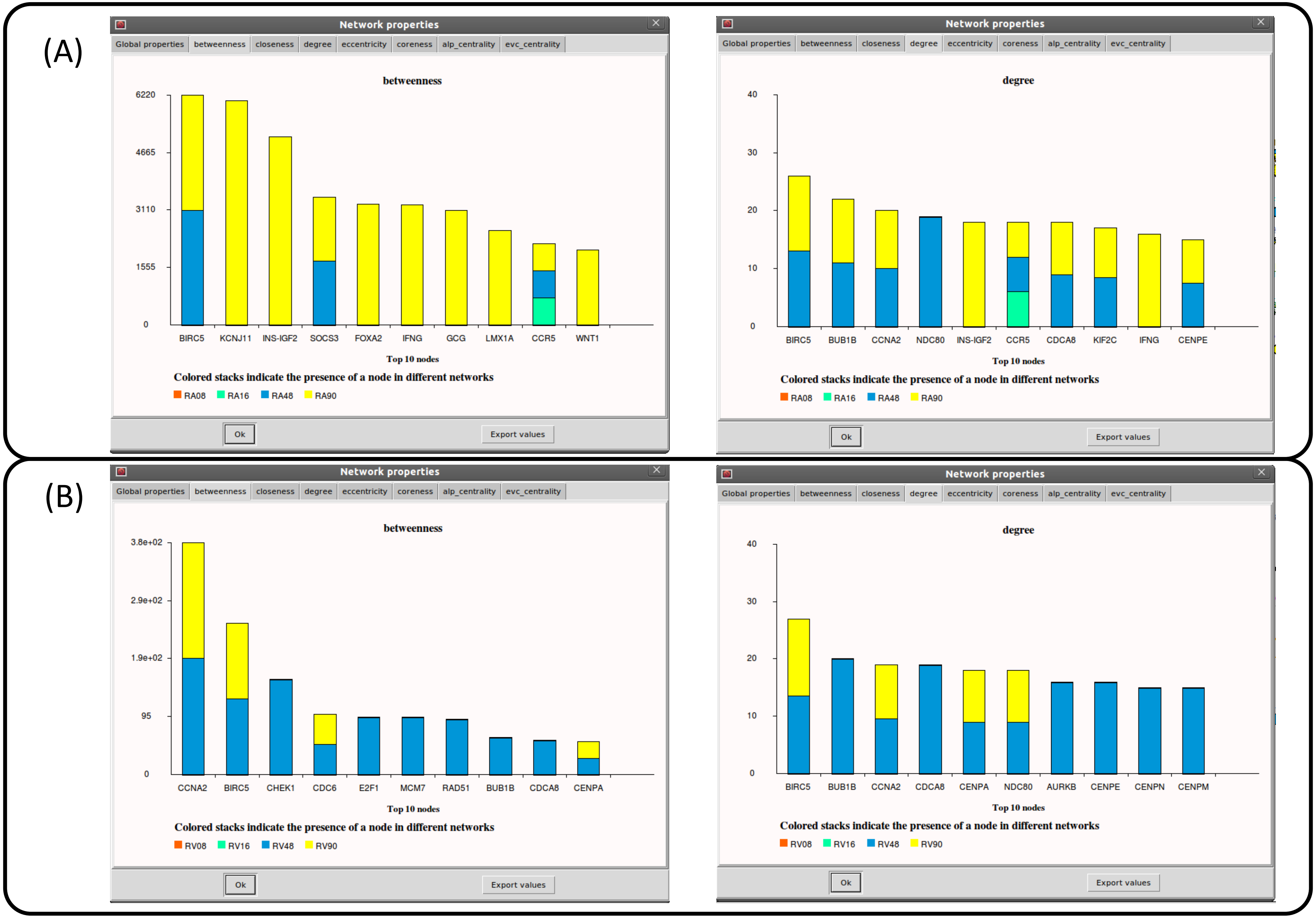

Figure S9. A view of the union of eight networks (RA8, RA16, RA48, RA90, RV8, RV16, RV48 and RV90), corresponding to host response to infections by two stains of *M. tuberculosis* (H37Ra and H37Rv), depicting the highly up-regulated genes at four different time-points (8, 16, 48 and 90 hours) post infection. CompNet has been used to highlight the betweenness of the nodes by mapping the node-radii to betweenness values. The presence of a node in a particular network (corresponding to the infection type/time-point) is also discernable from the colours of the 'pie-slices'. Nodes INS-IGF2 and SOCS3 appear to have very high betweenness and are seen to be present exclusively in the networks corresponding to infection by the H37Ra strain (at the 48th and 90th hour time-points).

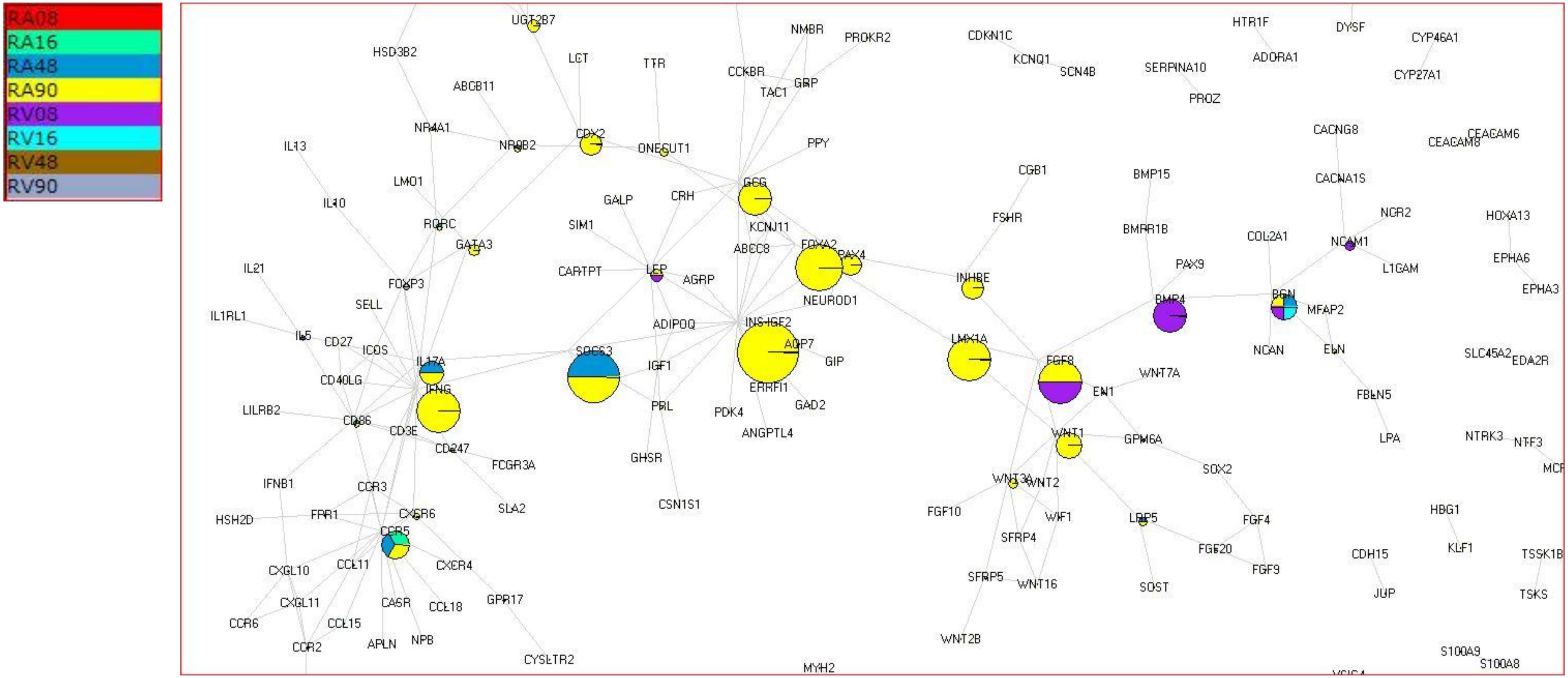

Figure S10. Graph properties of the union network, depicting upregulated host genes/proteins during infection by H37Ra and H37Rv strains of *M. tuberculosis*, are displayed as a cumulative bar plot. The top 10 nodes (in terms of the evaluated graph metrics) are shown in the respective bar plots using CompNet. The coloured stacks indicate the presence of the nodes in different networks. Nodes INS-IGF2, SOCS3 and CCR5 are identified to have the highest values of degree and betweenness. The stacks further indicate that these nodes are exclusive to networks corresponding to infection by the H37Ra strain.

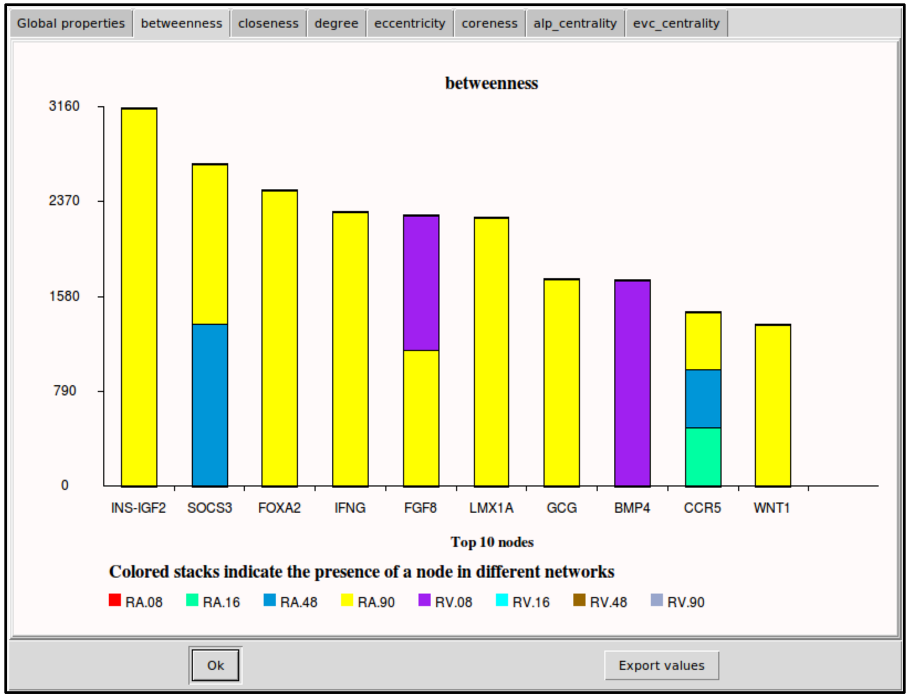

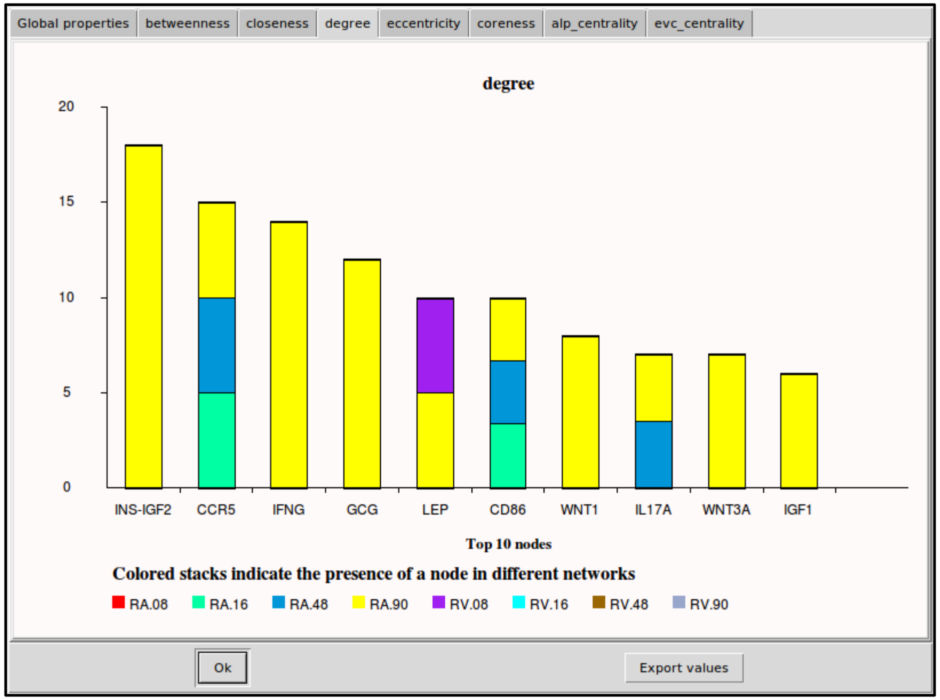

Figure S11. Shortest path analysis using CompNet. The numbers at the top represent the path lengths while those at the bottom represent the total number of shortest paths found in each network (colours corresponding to the selected networks as shown in the legend).

.

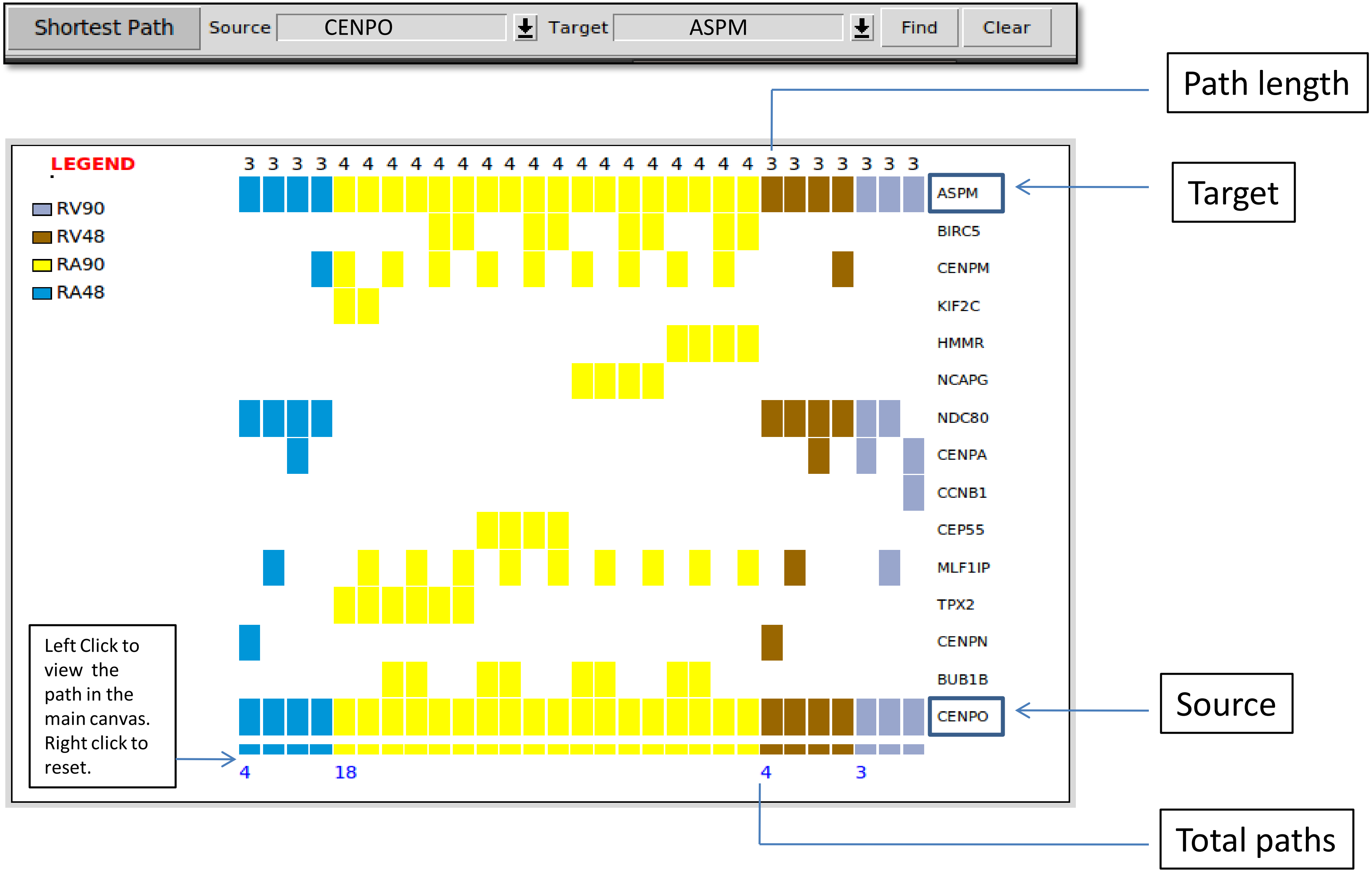

Figure S12. Visualization of community size distribution across different host response networks using CompNet. Most of the components belonging to the largest community C1 (represented as '1' in the figure) were seen to be perturbed at the 48th hour time-point during both H37Ra and H37Rv infection (RA48 and RV48). It may also be noted that the total number of interactions involving differentially regulated genes reduces in H37Rv infected cells at the 90th hour time-point. In contrast, for the H37Ra infected cells, the total number of interactions involving perturbed genes further increases at this late time-point post-infection, with a major contribution from the communities C2 and C3.

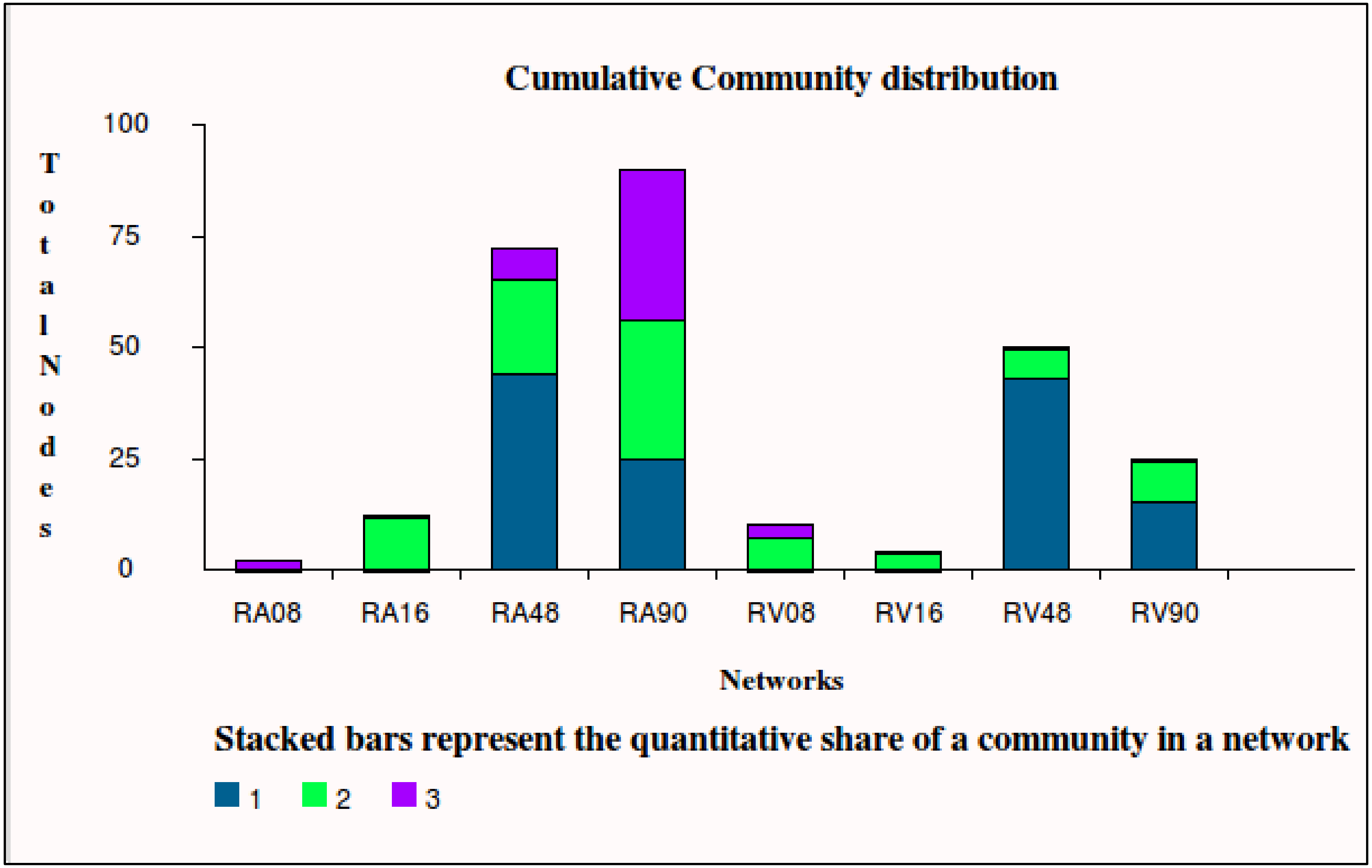

Figure S13. Visualization depicting intersecting edges between host response networks RA48 and RV48 (corresponding to significantly up-regulated and down regulated genes at 48 hours post host infection by H37Ra and H37Rv strains of *M. tuberculosis*) on the CompNet canvas. The union network is hidden in the current view for convenience of the reader. Majority of the intersecting edges belong to the community C1 (designated as '1' under the 'Communities tab' and identified by a distinct color).

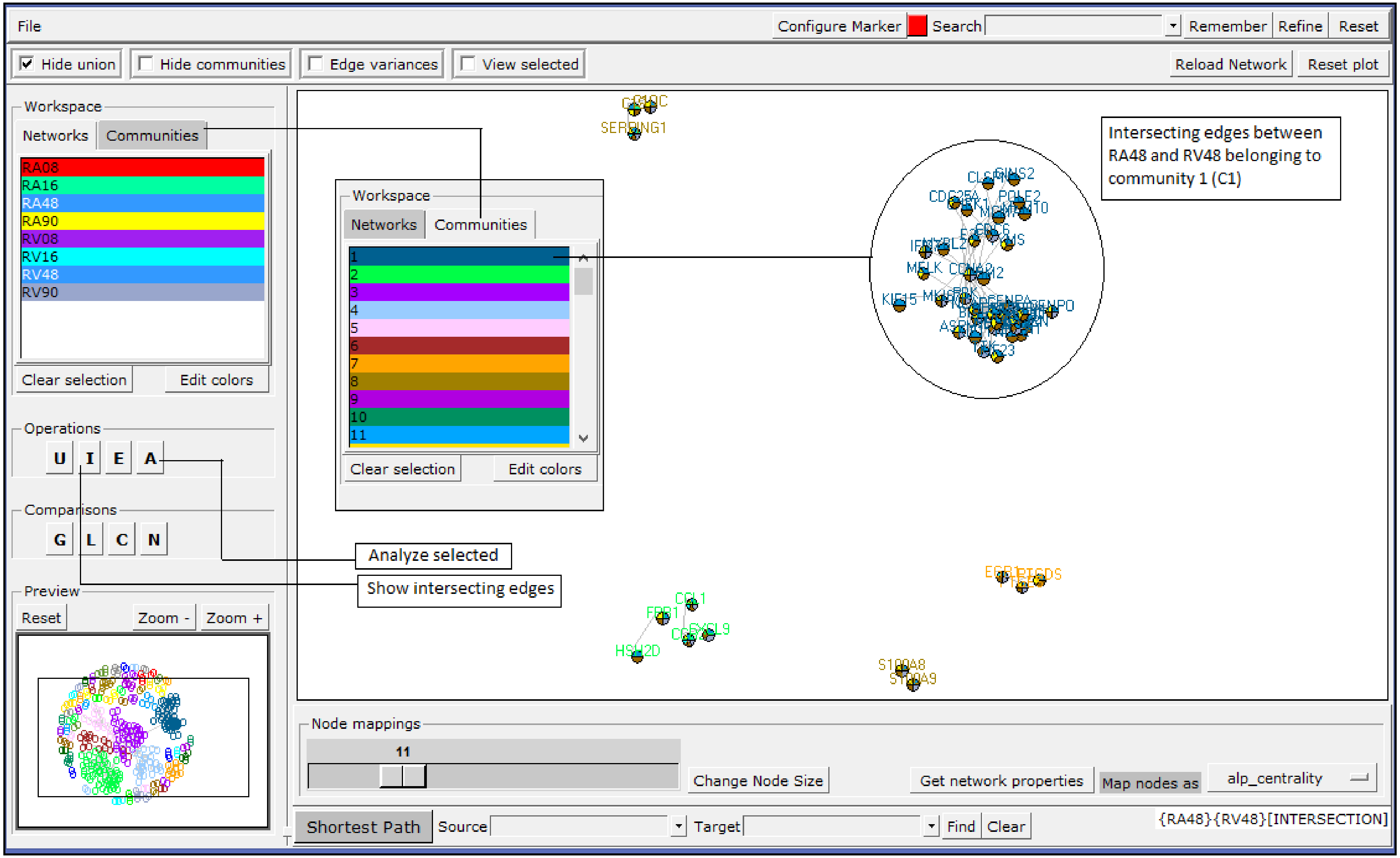

Figure S14. Visualizing 'edge-pie plot' for community C1 (please refer to main manuscript) using CompNet. Each pie-chart in the plot represents an edge (between the nodes identified on the two axes) where the pie slices represent the presence of the edge in different networks.

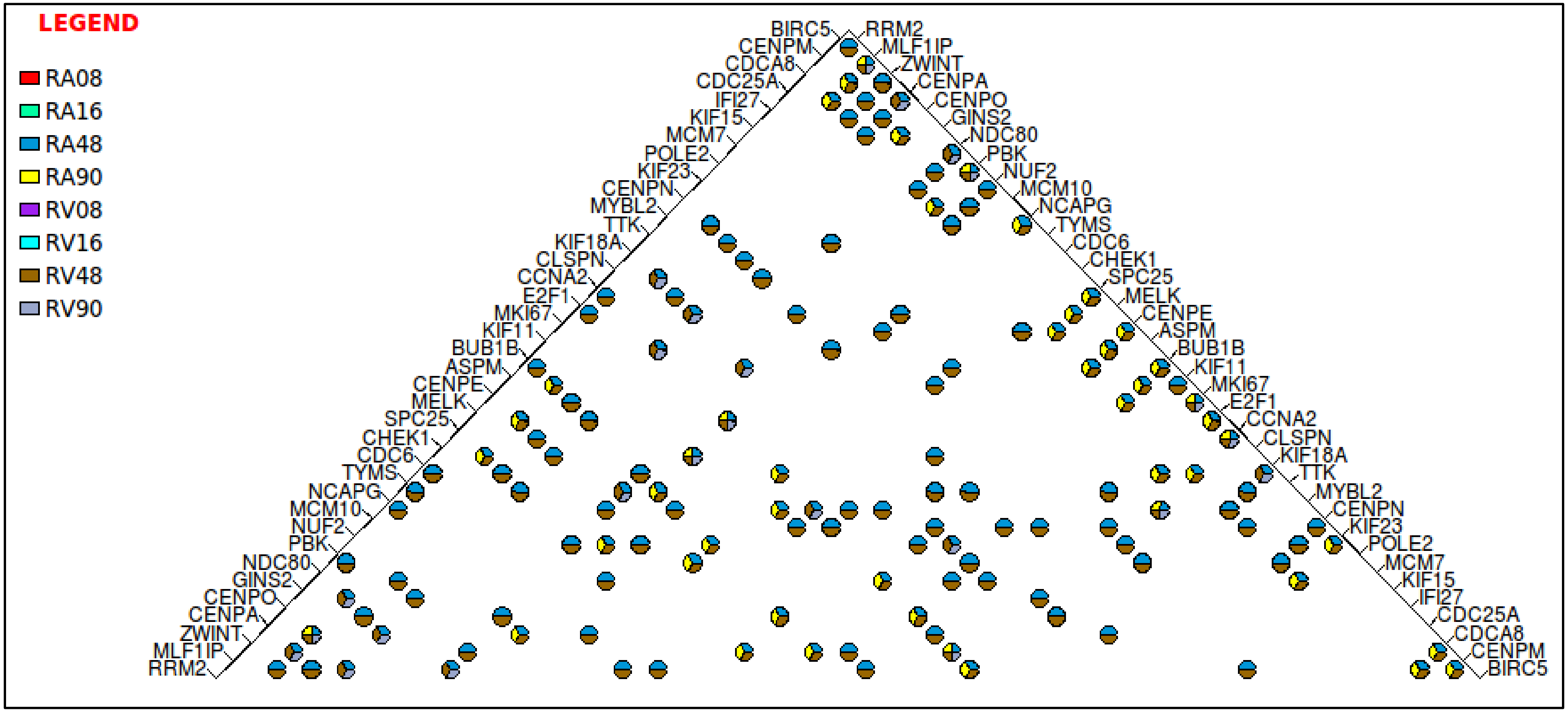

Figure S15. CompNet neighbour similarity index based similarity matrix and the corresponding dendrogram (tree) generated for the compared networks (RA8, RA16, RA48, RA90, RV8, RV16, RV48 and RV90), corresponding to the host response to infections by two stains of *M. tuberculosis* (H37Ra and H37Rv), constituted of the highly perturbed genes at four different time-points (8, 16, 48 and 90 hours) post infection. Networks RA48 and RV48, both corresponding to late infection time-points, are the most similar networks (which clusters together in the tree). However, the other late infection time-points, represented by the network RA90 and RV90, appears to be sufficiently different, and have branched separately.

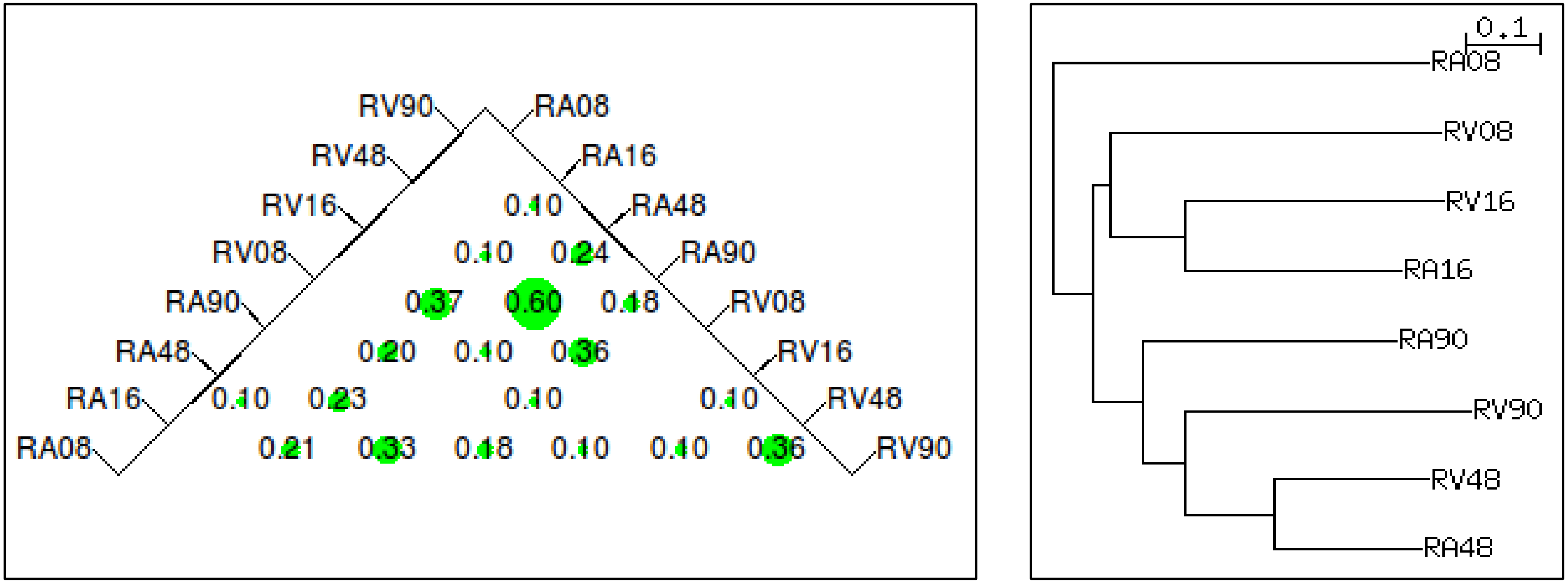

Table S1. Summary of network properties: (a) union of host response networks (all time points) pertaining to Mtb H37Rv infection, (b) union of host response networks (all time points) pertaining to Mtb H37Ra infection, and (c) union of all host response networks (all time points) pertaining to both types of infection. To check whether these properties were significantly different from that of a randomly generated network (having similar size), CompNet was used to generate 10,000 random networks, for each case, by randomly drawing nodes from the STRING human protein-protein interaction network. On comparison against the mean value of the network properties computed for the 10,000 randomly drawn networks, it was observed that the density, clustering co-efficient, and average path-lengths of the all the host response networks had significantly higher values ($p<0.05$).

(a)	Network property	Host response network against Mtb H37Rv infection (nodes = 146)	Random networks (10,000) of equal size drawn from STRING (mean)
	Network Density	0.028	0.001
	Clustering Co-efficient	0.621	0.151
	Avg. Path Length	2.418	1.42

(b)		Host response network against Mtb H37Ra infection (nodes = 285)	Random networks (10,000) of equal size drawn from STRING (mean)
	Network Density	0.012	0.001
	Clustering Co-efficient	0.493	0.256
	Avg. Path Length	5.282	2.273

(c)		Overall host response network (nodes = 358)	Random networks (10,000) of equal size drawn from STRING (mean)
	Network Density	0.01	0.001
	Clustering Co-efficient	0.501	0.263
	Avg. Path Length	5.808	2.867

Table S2. GO biological process terms which are found to be significantly (P < 0.001) enriched in the communities C1, C2 and C3. Only those GO terms which are associated to >25% of the nodes (genes/proteins) of the respective communities are listed.

Community 1	Community 2	Community 3
Cell cycle	Immune response	Cell surface receptor linked signal transduction
Cell cycle process	Cell surface receptor linked signal transduction	Intracellular signaling cascade
Mitotic cell cycle	Defense response	G-protein coupled receptor protein signaling pathway
Cell cycle phase	G-protein coupled receptor protein signaling pathway	Regulation of secretion
M phase	Behavior	Cell-cell signaling
Mitosis	Response to wounding	Response to organic substance
Nuclear division	Taxis	Regulation of cellular localization
M phase of mitotic cell cycle	Chemotaxis	Behavior
Organelle fission	Locomotory behavior	Homeostatic process
Cell division	Inflammatory response	Regulation of apoptosis
Microtubule-based process	Homeostatic process	Regulation of programmed cell death
Regulation of cell cycle	Regulation of cell proliferation	Regulation of cell death
		Neurological system process
		Positive regulation of developmental process
		Regulation of system process
		Response to endogenous stimulus
		Chemical homeostasis
